# How do policies promote the sustainable development of older-adult care industry? A configuration analysis based on policy tools

**DOI:** 10.3389/fpubh.2024.1430679

**Published:** 2024-10-11

**Authors:** Xiangwei Zhang, Zhengnan Lu, Dongdan Zhu, Yuting Zhang

**Affiliations:** ^1^School of Management, Jiangsu University, Zhenjiang, China; ^2^Jingjiang College, Jiangsu University, Zhenjiang, China; ^3^Department of Mechanical and Manufacturing Engineering, University of Calgary, Calgary, AB, Canada

**Keywords:** older-adult care industry, policy tool, policy mix, qualitative comparative analysis, text mining

## Abstract

**Background:**

Under the background of population aging in China, the demand for older-adult care services and products is growing, and the older-adult care industry has great development prospects. A sound older-adult care policy system, that is, an effective policy tool mix, plays an important role in improving the sustainable development of older-adult care industry.

**Materials and methods:**

Based on older-adult care policy documents from 31 Chinese provinces, this research extracts older-adult care policy tools via text mining. Then extracted policy tools are taken as conditional variables, and the development of older-adult care industry, which is manifested by the number of older-adult care companies across 31 regions is taken as the result variable. Through applying qualitative comparative analysis, the combined effect of different policy tools on the development of older-adult care industry is obtained.

**Results and discussions:**

Results show that a single policy tool cannot constitute the necessary condition to facilitate the older-adult care industry. Hence, policy tools should be applied in combination. Five sustainable policy tool mixes which can promote the development of older-adult care industry are summarized, namely supporting policy-driven mode, fiscal and tax support mode led by supply-oriented policy tools, double-team mode driven by fiscal and tax support and the consumer market, multi-subject joint force mode, and technology compensation mode. The overall findings of this study imply that exploring the policy tool combinations is of vital importance to the sustainable development of older-adult care industry.

## 1 Introduction

With rapid economic development and continuous improvement in health care, China entered an aging society in 1999, and societal aging continues to intensify. The results of the seventh national census indicate that the population aged 65 and above numbers 19.064 billion, accounting for 13.5 percent of the total population (1). Population aging can represent the progress of social civilization to a certain extent, while it also places enormous pressure on socio-economic elements. According to the Research Report on the Trends of Population Aging in China, the proportion of population over 80 years of age to the total population is projected to reach 21.8% in 2050 owing to the impact of the baby boom in the early 1960s and 1970s (2).

Given the current situation of accelerating aging, traditional older-adult care mode which is supported by families and society has difficulty in meeting the diversified and personalized demands of older adults. The large-scale demand for quality older adult care exceeds China's existing older adult care service supply capabilities (3). In response, the development of older-adult care industry can enhance the provision of products and services capable of satisfying the older adults' needs. Developing older-adult care industry is an important strategy to solve the contradiction that the demand for older-adult care services is growing but the provision of older-adult care services is seriously lagging behind. Since the Healthy China Strategy was put forward, all regions in China have been striving to seize development opportunities in the older adult care industry (4). Specifically, the term “older-adult care industry” refers to a market-oriented economic group comprising companies whose target customers are older adults, and these companies encompass all commodity producers and service providers concerned with satisfying the older adults' physical and spiritual needs (5).

With the deepening trend of aging and the declining birthrate, developing the older-adult care industry has become a matter of concern for all sectors of society and governments. Notably, it is a common knowledge that a sound and strict policy system is conducive to the sustainable development of the older-adult care industry (2). Hence, the key to promoting the sustainable development of older-adult care industry is formulating a scientific and sustainable policy system. Policy tools are the elements that constitute the policy system. They are a series of specific measures that governments utilize to achieve policy objectives and values (6). Older-adult care policy system is constructed through a reasonable combination of different policy tools. Optimizing the overall layout of older-adult care policy tools and forming a scientific and effective policy tool mix are of vital significance to support the sustainable development of older-adult care industry and promote the high-quality development of China's economy. Specifically, this paper focuses on the questions as follows:

- Which policy tools are included in the older-adult care policy system in China?
- Can a certain policy tool facilitate the development of the older-adult care industry?
- Which policy tools should be applied in combination to stimulate the development of older-adult care industry?

The answers to these questions are still unclear. Existing research on the older-adult care policy mainly place emphasis on analyzing the characteristics of policies taking into account national conditions (7–9) and excavating policy texts through quantitative methods (5, 10, 11). Research on the configuration analysis of various older-adult care policy tools is very lacking. Therefore, taking the above problems as an entry point, the current paper aims, from a holistic research perspective, to reveal a beneficial and sustainable combination of policy tools and figure out how the configuration of different policy tools may enhance the development of older-adult care industry. To fulfill these objectives, the current research samples 31 representative regions and their older-adult care related policy texts. Policy tools are extracted among the sampled policy texts through text mining technology, and the extracted policy tools are taken as conditional variables. The number of older-adult care related enterprises in 31 regions which can represent the development of older-adult care industry is taken as the result variable. Then we adopt qualitative comparative analysis (QCA) to investigate the combined effect of policy tools on the older-adult care industry, offering policy implications to optimize China's older-adult care policy system.

The main contributions of this paper are reflected in the following two aspects.

- In terms of research content, the current research investigates the older-adult care policy tool mix based on the understanding that different arrangements and combinations of various policy tools will produce different policy effects. Hence, to optimize the overall layout of older-adult care policies and enhance the sustainable development of older-adult care industry, this paper focuses on the combined effect of policy tools on the older-adult care industry.
- In terms of research methodology, this study combines text mining technology with QCA method, and introduces the combination into the research on older-adult care policy tool mix. With the support of text mining technology, this study explores the internal associations of unstructured policy texts and extracts scientific policy tools from policy texts. Based on the results of text mining, QCA is applied to exploit its advantages in dealing with the nonlinear relationship of multiple conditional variables (12). In this research, QCA reveals the relationship between policy tools and the development of older-adult care industry.

The rest of this paper is organized as follows. Section 2 reviews the relevant literature pertaining to the older-adult care industry, older-adult care policy and QCA. In Section 3, we introduce the research methodology and data sources. Section 4 is dedicated to identifying the conditional variables through text mining technology and determining the result variable, as well as the variable assignment. Section 5 presents the application of csQCA method to investigate the older-adult care policy tool mix. Conclusions and policy suggestions are put forward in Section 6.

## 2 Literature review

This study attempts to apply the combination of text mining technology and QCA method to explore the configuration of various older-adult care policy tools. The literature related to this paper can be classified into three groups: older-adult care industry, older-adult care policy and QCA.

### 2.1 Older-adult care industry

The population is aging in tandem with the miniaturization of families and a declining birth rate. Hence, developing the older-adult care industry has become one of vital strategies to deal with the aging problem. In terms of the future development trend of the older-adult care industry, it is important to highlight that smart older-adult care industry is gaining momentum under the digital economy and digital technology, characterized by cloud computing, artificial intelligence, big data, and blockchain. For example, Valdivia et al. (13) propose that information technologies have made it possible to promote the use of IoT, and one of the industries with greatest challenges is the health sector. They design a low-cost digital vital signs capturer for older adults, facilitating the development of smart older-adult care industry. Maresova et al. (14) present the current trends and possibilities in applying smart information and communications technology (ICT) solutions for in-home care concerning diseases in old age, and propose the gap between scientific publications and actual solutions of smart older-adult care industry. In addition to the smart older-adult care, there are multiple sub-industries regarding the older-adult care industry, such as older-adult finance (3, 15, 16), older-adult tourism (17, 18), older-adult insurance (19–21), and older-adult real estate (22).

Specifically, in terms of older-adult finance, there are increasing reports of older adult financial exploitation during the COVID-19 pandemic as many older adults are unfamiliar with the digital technologies and vulnerable to be targeted by perpetrators. Therefore, older-adult finance is of vital significance in the older-adult care industry. Zhang et al. (3) investigate trends in usage of electronic financial technologies by older adults and describe new technology-based mechanisms of older adult financial exploitation. Neuman et al. (15) examine the status of older women in 12 industrialized nations and argue that the aging of the U.S. population presents challenges in financing care and meeting the health care needs of older Americans, promoting the advancement in the older-adult finance. Additionally, older adults have become important target customers for the leisure tourism market on a global scale (23). Stoncikaite (17) proposes that the older adults, especially the representatives of the baby-boom generation, account for a large share of all holiday spending, and older-adult tourism becomes the greatest driving force in the hospitality tourism. Kim and Kang (18) focus on the older adults' user experience of virtual tourism and prove the applicability and effectiveness of VR tourism in the older adults. Health insurance is also a prospecting section in the older-adult care industry. Alaazi et al. (19) explore the health insurance exemptions for older adults in Ghana and propose that this insurance exemption policy can ensure the free healthcare for older adults at publicly-funded facilities in Ghana. Golomski (20) also explores the private health insurance in South Africa from the perspective of race-class. To enhance the health insurance literacy of older adults, McCormack et al. (21) develop an instrument to measure the familiarity with health insurance terminology and proficiency with the Medicare program. Real estate is also a domain of international discussion in the older-adult care industry. Van Hoof and Boerenfijn (22) highlight that enabling an older adult to age-in-place requires new housing arrangements. They take De Benring in Voorst, The Netherlands as the study case, and reinvent existing real estate of social housing for older adults.

Researchers also pay attention to the current challenges of older-adult care industry. It is concluded that the insufficient market (24, 25), low investment (26, 27), lack of professionals (28, 29), poor technology (2) are the factors constraining the development of older-adult care industry. Especially, Zhang and Zhang (30) argue that the current challenge of older-adult care industry that China has been facing is the uneven development between the eastern regions and northwestern regions. In order to stimulate and enhance the development of older-adult care industry, various promotion paths and strategies are put forward, including constructing a sound policy system (31), cultivating the vitality of market entities (32), establishing an efficient industrial chain (33) and focusing on the personnel training (34).

In summary, academia has recognized the importance of older-adult care policy system in the sustainable development of older-adult care industry.

### 2.2 Older-adult care policy

As mentioned above, a scientific policy system is an important basis and foundation to ensure the sustainable development of older-adult care industry. Older-adult care policy system should be closely aligned with the older adult's needs and conform to new trends of older-adult care industry. Extant older-adult care policy research has mainly focused on the following two aspects.

- Firstly, different countries around the world analyze the characteristics of older-adult care policies taking into account their own national conditions. For example, to evaluate and analyze the local aging policies in Portugal, Bárrios et al. (7) construct the Model for Aging Local Policies Analysis to convert the active aging paradigm into a practical approach, identifying priorities for aging policies in two Portuguese communities. Through conducting interviewing, Doshmangir et al. (8) figure out several key factors influencing older-adult care policies in Iran, such as government financial support, policy structure, actors and stakeholders, etc. Mandville-Anstey et al. (9) conduct a critical document analysis of provincial aging policies in Newfoundland and Labrador, Canada, exploring aging women's notions of health and the body in relation to the aging process. Ng et al. (35) investigate the impact of aging policy in Singapore on societal age stereotypes and ageism, and conclude that the impact of policy on medicalization is stronger when a society is more aged. Martynova (36) pays attentions to the goal of active longevity in Russian policy documents on aging and takes active longevity as a policy goal, alongside healthy aging, employment, and social participation. Based on a three-dimensional ecological chain analysis model of the older-adult care industry, Wang et al. (37) summarized China's older-adult care policy's developmental pattern in three basic points, which are traditional transformation base point, mainstream growth base point, and frontier leading base point, respectively. Additionally, comparative analysis of older-adult care policies between different countries also draws the attention of researchers. For example, to compare Canada, Japan, and Korea in terms of the framing of policy challenges related to demographic aging, McDaniel (38) carries out the comparative analysis and case studies of aging polices. Calvo et al. (39) adopt comparative analysis and case studies to investigate the aging policies in Mexico, Costa Rica, Argentina, Chile, and identify the similarities and trends in the substance of historical and current aging policies across countries.
- Secondly, researchers in the field of policy study tend to adopt the quantitative research to excavate the older-adult care policy texts, exploring the evolution and development of older-adult care policy. For example, Feng and Nan (5) adopted text content analysis, co-word analysis, social network analysis, and clustering analysis to quantitatively examine older-adult care policy texts, and conclude the policy level, policy evolution, and practical effectiveness. Dizon et al. (10) collect eleven global, national, and local aging policies and apply thematic and discourse analysis to explore the development of older-adult care polices and answer the question of “What is meaningful participation for older adults?” Li et al. (40) adopt a quantitative analysis based on text mining to explore the priorities and instruments of local older adult care policies in China, and conclude that the most important policy instruments are regulatory systems, financial services, and strategic guidance.

To sum up, the existing studies have paid attention to the older-adult care policies. However, the configuration and combined effect of different policy tools is still an open question.

### 2.3 Qualitative comparative analysis

Qualitative Comparative Analysis (QCA) method was initially applied to address political, diplomatic, and societal issues. However, its integration into management studies gained significant attraction in recent years (12). Fiss's publications in 2007 and 2011 extensively merged QCA with management studies, receiving formal recognition from the management community (41). Over the following years, there was a surge in global publications utilizing QCA, particularly notable in the last three years. Presently, QCA has permeated various facets of management studies, including supply chain management (42), business management (43), environmental governance (44), and social problems (45, 46). For example, Stekelorum et al. (42) apply the fuzzy-set QCA to explore the different combinations of internal and external green supply chain management practices that lead to enhanced operational and financial performances in third-party logistics providers. Warren et al. (45) use QCA to explore the causal pathways to reduced bullying in a whole-school intervention. To answer the question of what drives university engineering students to become entrepreneurs, Rippa et al. (43) apply QCA with a sample of 10,008 engineering students in 10 European countries to investigate how configurations of factors lead to the entrepreneurial intention. In the field of environmental governance, Liu et al. (44) investigate the interactions of conditions that affect the intergovernmental collaboration on the air pollution control through QCA. Based on a fuzzy-set qualitative comparative analysis, Hastings (46) explores how financial stress, poor health or crisis events interact with emotional wellbeing to influence housing security for Indigenous Australian families living in poverty.

In sum, academia has recognized the importance of older-adult care policy, and profound research has been conducted on the older-adult care industry in the form of policy explorations. However, the following questions remain unanswered: What specific policy tools does older-adult care policy system include? What is the combined effect of various policy tools on the development of older-adult care industry? To contribute to filling this research gap, the current research adopts QCA to investigate older-adult care policy tool mix modes. Employing QCA method to explore the older-adult care policy not only serves as a complementary and expansive means for policy research from a novel perspective, but also ameliorates the inadequacies of QCA method implementation in the realm of policy research in modern-day Chinese management studies.

## 3 Methods and data

### 3.1 Methods

#### 3.1.1 Text mining

The current research adopts text mining to analyze older-adult care policy texts to achieve the immediate goal of obtaining more scientific policy tools and the overarching goal of conducting configuration analysis of policy tools. Text mining combines information retrieval and machine learning. Its main purpose is extracting information from large amounts of text data to satisfy users' needs. Text mining can be employed to explore an original text's potential rules and help users extract valuable information (47). Given that older-adult care policy texts are lengthy and complex, relying solely on human labor to peruse such content comes with a huge workload and is susceptible to subjectivity. Text mining technology can overcome these problems and assist in the extraction of comprehensive older-adult care policy tools. Therefore, the current research takes the older-adult care policies of 31 Chinese provinces, autonomous regions, and municipalities as the original corpus for text mining. After preprocessing, which entails removing stop words and loading a custom dictionary, word segmentation is performed. The segmented policy texts are then subject to K-means clustering. Next, the TF-IDF algorithm is applied to obtain keywords for each cluster, based on which each cluster's core phrases are extracted. Finally, the older-adult care policy tools can be obtained by summarizing each cluster's characteristic.

#### 3.1.2 Qualitative comparative analysis

QCA is a widely used social science research paradigm. It is a research method based on Boolean algebra and set theory (48). QCA is case oriented. Each case is represented as a combination of causal and outcome conditions. QCA assumes that the cause of a certain phenomenon is complex and nonlinear, and the phenomenon is therefore the result of a combination of multiple condition configurations (49). The fundamental concept of QCA is that cases can be expressed by logical statements in which the conditional variables (independent variables) for each case, in combination, are regarded as logically implying the score on the result variable (dependent variable) for that case. The QCA method includes three operation modes: fuzzy set QCA (fsQCA), clear set QCA (csQCA), and multi-value QCA (mvQCA).

The current research adopts csQCA, and the reasons are as follows. First, in csQCA, the conditional and result variables are both binary, which means each variable's value is either zero or one. Considering the aim of the current paper, that is, investigating the complex influence of policy tool configuration on the older-adult care industry, older-adult care policy tools are taken as the conditional variables, and the number of older-adult care related enterprises is taken as the result variable to reflect the development of older-adult care industry. With respect to the assignment of conditional variables, it is only necessary to judge if a certain policy tool exists in each case. There is no need to count the amount of the specific policy tool in each case, thus a value of either zero or one is assigned to each policy tool. Additionally, each case's result variable is assigned with a value of zero or one based on the number of older-adult care related enterprises in that case compared with the average level. Therefore, it's convenient and expeditious to employ csQCA to explore the older-adult care policy tool mix. Second, the csQCA method generally requires 10 to 60 samples (50), which is sensible for small and medium-sized case analysis. The current research samples 31 Chinese regions, and it meets the sample requirements of csQCA. Generally, csQCA includes the following core steps: (1) selecting research samples, (2) determining conditional and result variables, (3) assigning values to variables and constructing a truth table, (4) conducting the necessity analysis and sufficiency analysis.

**Figure 1:**
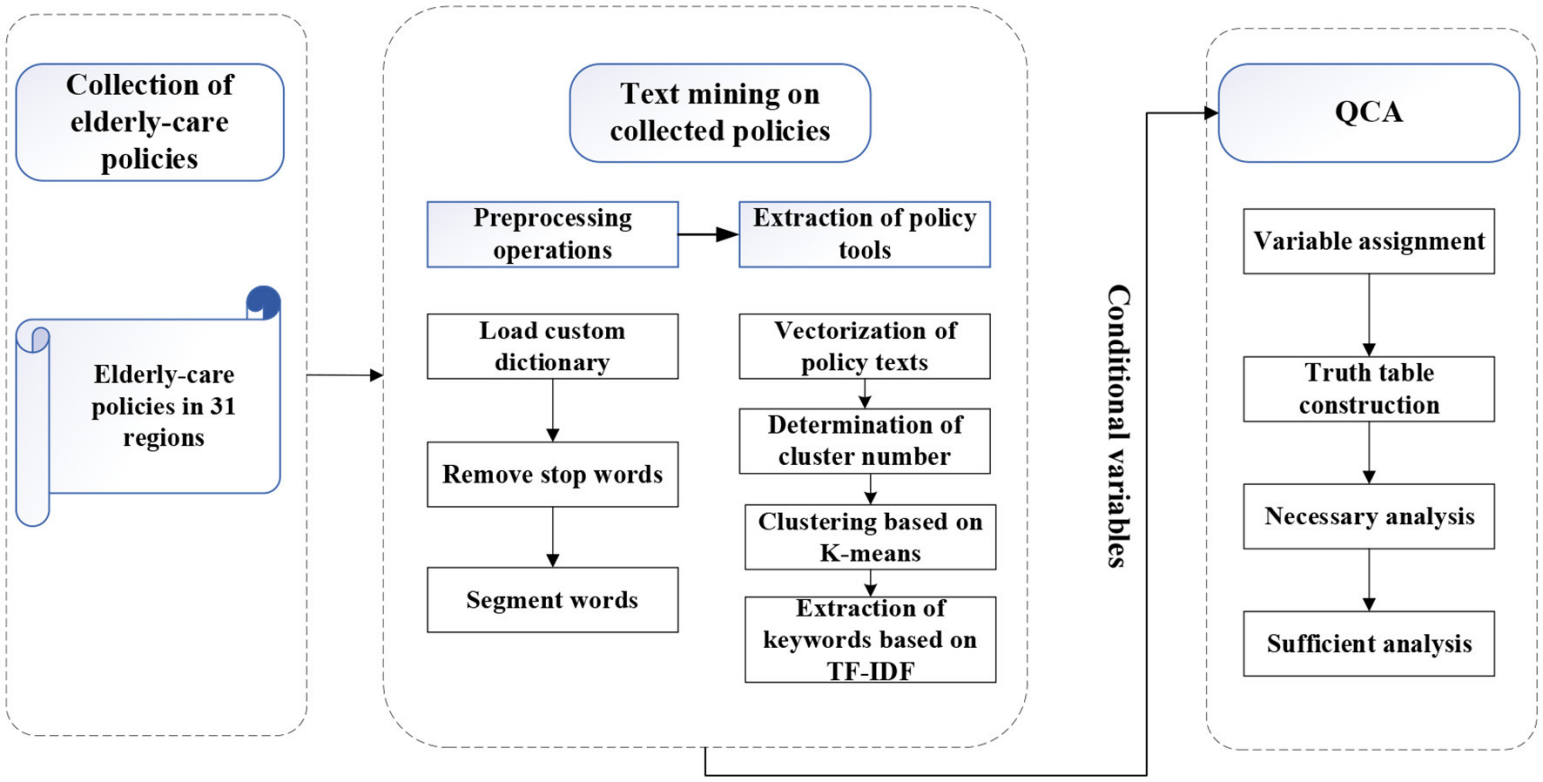
Framework of exploring older-adult care policy tool mix.

The overall framework of exploring older-adult care policy tool mix based on the method of text mining and QCA is illustrated in Figure 1 and includes two main components. The first part is dedicated to extracting older-adult care policy tools among older-adult care policies by means of text mining. In the second part, the extracted policy tools are taken as the conditional variable, and the number of older-adult care enterprises which reflects the development of the older-adult care industry is taken as the result variable. The configuration analysis is carried out through QCA and the combined effect of policy tools on older-adult care industry is obtained.

### 3.2 Data

Since the policy effect generally has a time lag of 2 years, the current research selects older-adult care policies issued by 31 Chinese regions before 2020. To exhaust the older-adult care policies and collect the comprehensive and detailed policy texts, we take the official government websites and the China Older-adult care website as data sources of current paper. Additionally, the legal inquiry software PKULAW is also incorporated into the current research as an important data source. We can collect older-adult care policies by retrieving keywords and browsing relevant laws and regulations on the software PKULAW and local government websites. Notably, older-adult care related policies, regulations, and relevant opinions can often be found in other topics, such as tourism, environment, technology, finance, etc. Therefore, in addition to a precise search with “older-adult care industry” as the keyword, policy texts with keywords or themes such as “older-adult care,” “pension,” “aging,” and so forth, are also worthy of attention. To obtain a comprehensive older-adult care policy text, “full text” and ambiguous retrieval rules should be applied when searching policies via keyword retrieval. To eliminate the interference of irrelevant policy information and improve the accuracy of text mining, only texts related to the older-adult care industry comprise the original corpus to be mined. For example, although the document “Implementation Opinions of Shanxi Province Government on Promoting Reform of Tourism Industry” is a special tourism policy, it also includes the texts about promoting the development of the older-adult care industry. We just take into account the texts pertaining to the development of older-adult care industry and incorporate them into the original corpus. The current research ultimately collects a total of 132 older-adult care policy texts with respect to the development of older-adult care industry from 31 regions.

## 4 Research variables

### 4.1 Conditional variables

#### 4.1.1 Text preprocessing

**Figure 2:**
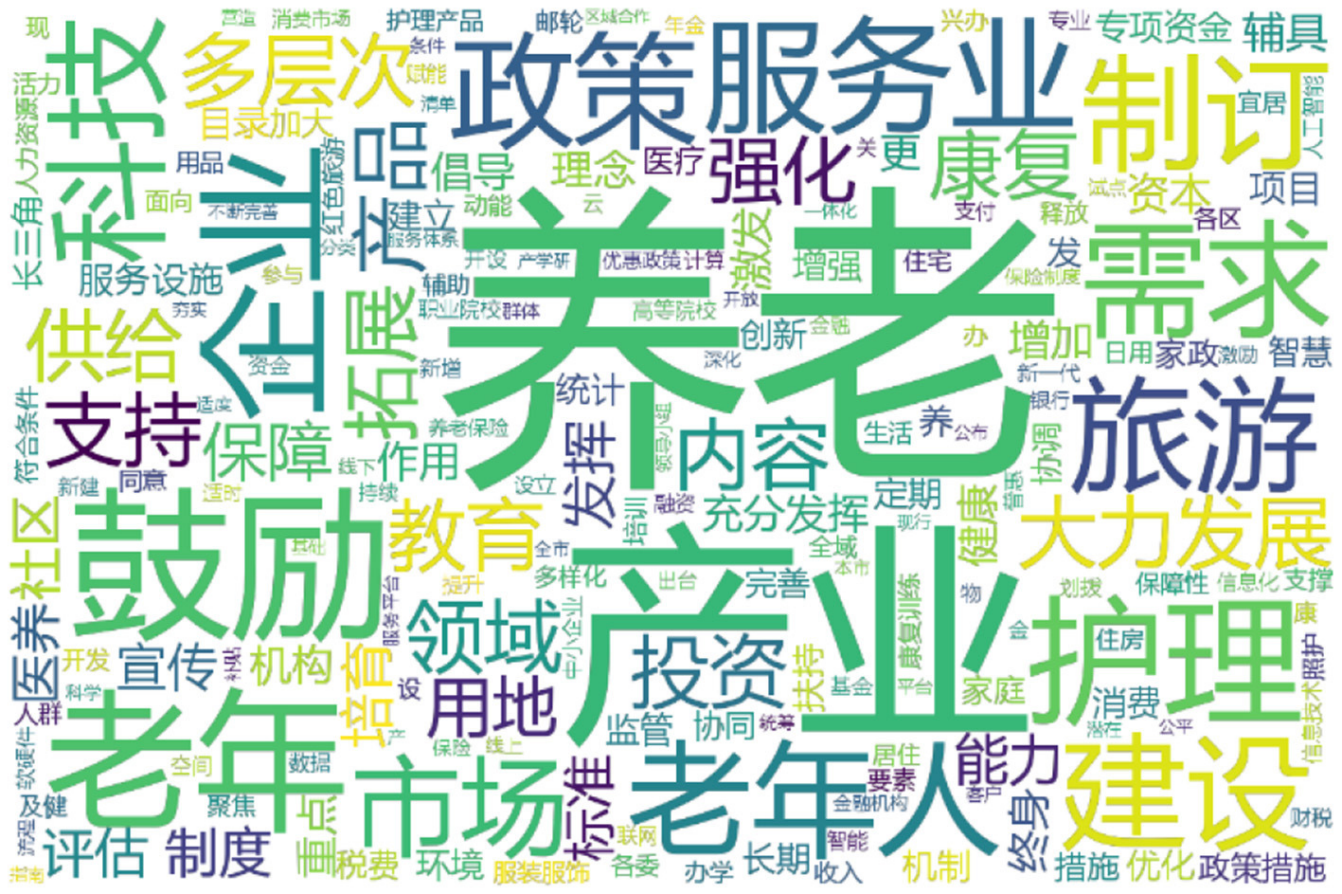
Word cloud of older-adult care policy texts.

Text preprocessing is the first step in text mining. This study utilizes Python and the third-party library *jieba* to perform word segmentation and generate word frequency statistics for 132 older-adult care policy texts. Before word segmentation, stop words are removed, and a custom dictionary is loaded. The current research uses Harbin Institute of Technology's stop word dictionary to remove meaningless words. To improve the accuracy of text mining and eliminate the interference of words that commonly used in the older-adult care industry, the stop word dictionary included words such as “development,” “implementation,” “country,” etc., and a customized older-adult care industry dictionary is constructed with the aid of *Sogou Cell Dictionary*. Next, precise mode in *jieba* is used to perform word segmentation, after which the *WordCloud* function is adopted to generate the word cloud of older-adult care policy texts, as shown in Figure 2. In Figure 2, the large font size used for words such as “older-adult care” and “industry” indicates that those words are frequently used in older-adult care policy, suggesting that the collected policy texts are closely related to the subject of older-adult care industry.

#### 4.1.2 Text clustering via K-means

In this study, to collect policy texts comprehensively, both the accurate retrieval of “older-adult care industry” from the title and the fuzzy retrieval of the same term from the full text are adopted. Based on these retrieval rules, the data sources are diverse, and the collected policy texts are considerably complex. Hence, to extract older-adult care policy tools from such complicated texts, the current research clusters older-adult care policies via K-means cluster analysis. Text clustering via K-means is an unsupervised machine learning method that does not require training or the pre-categorization of texts. K-means clustering analysis is carried out based on text similarity. The main steps of categorizing the older-adult care policy texts through K-means clustering analysis are as follows:

- Step 1: Vectorization of policy texts. Word segmentation and word frequency statistics are the basis of K-means clustering analysis. However, word frequency statistics cannot be directly modeled through K-means clustering analysis. In this study, further vectorization of policy texts is needed, that is, constructing a word frequency matrix. The specific operations of vectorization in the present research are as follows. Firstly, the *Coutervectorizer* package is imported from the *sklearn* library, and then instantiation is performed. Secondly, to vectorize the policy texts and obtain the word frequency matrix, the command *count.fit_transform* is applied to the word segmented texts.
- Step 2: Determining the number of clusters. The elbow method is employed to determine the optimal category number *k* in K-means clustering analysis. The core indicator of the elbow method is the sum of squares of errors (SSE). When the value of *k* is smaller than the real cluster number, the SSE will drastically decrease because the increase of *k* will amplify each cluster's aggregation. When the value of *k* is close to the real cluster number, the SSE will decrease to a much lesser extent, and its value trend tends to be flat with the continuous increase in *k*. Therefore, the graph depicting the relationship between SSE and *k* is elbow-shaped, and the *k* value that corresponds to the position of the elbow joint is the final category number *k* in the K-means clustering analysis (51). Equation 1 is the formula for calculating the SSE.
(1) $$\begin{array}{l} SSE=\overset{k}{\underset{i=1}{\sum }}\underset{p\in C_{i}}{\sum }{\left|p-m_{i}\;\right|}^{2} \end{array}$$
  where *C*_*i*_ indicates the i^*th*^ cluster, *p* is the sample point of *C*_*i*_, *m*_*i*_ is the centroid of *C*_*i*_, and *SSE* is the clustering error of all samples, representing the clustering effect. In this study, with the help of Python software and the application of the elbow method, an obvious turning point is found on the SSE curve when *k* = 9. Therefore, the collected older-adult care policy texts are divided into nine categories.
- Step 3: Clustering analysis. The *KMeans* package is imported from the *sklearn.cluster* library. Next, based on the word frequency matrix of older-adult care policy texts obtained in Step 1, model instantiation is carried out, with nine as the parameter of *n_clusters*. The *fit* interface is used to perform clustering analysis on the word frequency matrix. Results show that the number of policy texts corresponding to each category is 32, 26, 22, 18, 12, 8, 5, 5, and 4, respectively.

#### 4.1.3 Extracting policy tools via TF-IDF algorithm

The TF-IDF algorithm is adopted to obtain keywords for each cluster. To clarify the theme and further summarize the policy tool of each cluster, the following operations are executed: (1). The policy texts are classified and integrated into nine policy texts according to the corresponding category and (2) the TF-IDF algorithm is used to extract keywords for the nine policy texts. Compared with word frequency statistics, the TF-IDF algorithm offers word distinction and criticality. Nine policy documents are imported into the *jieba.analyse* library. After removing the stop words, the command *jieba.analyse.extract_tags* is applied to perform word segmentation and calculate the TF-IDF value for each segmented word. As a result of the above operations, the top 10 keywords sorted by TF-IDF value in nine policy categories are shown in Table 1.

**Table 1:** Clustering analysis results and keyword extraction.

**Cluster 1**	**Cluster 2**	**Cluster 3**	**Cluster 4**	**Cluster 5**	**Cluster 6**	**Cluster 7**	**Cluster 8**	**Cluster 9**
Products for the older adult	Smart older-adult care	Finance	Medical care	Professional	Policy	Demonstration	Insurance	Land use
Nursing	Intelligence	Fund	Cure	Talents	Regulation	Focal point	Inclusive	Land supply
Service	Big data	Tax revenue	Medical care and nursing interaction	Post	Environment	Older-adult care	Finance	Cost
Older-adult care institutions	Smart older-adult care	Special	Older-adult care	Older-adult care	Older-adult care	Leading enterprise	Subsidy	Expand
Home care	Informatization	Discount	Recovery	Skills	Support	Guide	Consumption	Demand
Accessories	Product	VAT	Nursing	Training	Detailed rules	Brand	Demand	Older-adult care
Products	Internet	Capital	Institution	Level	Project	Project	Stimulate	Guarantee
Community	Older adult	Enterprise	Integrated	Personnel	Supervision	Community	Profit	Land price
Travel	Demand	Support	Hospital	School	Supporting	Cultivate	Universally beneficial	Enterprise
Education	Nursing home	Older-adult care	Older adult	Education	Statistics	Lead	Nursing	Institution

According to the K-means clustering results, Cluster 1's keywords include “products for the older adult,” “nursing,” “service,” “accessories,” “home care,” etc. Therefore, Cluster 1 is labeled as “older-adult care products and services.” Policy texts that fall under the policy tool of “older-adult care products and services” emphasize that enhancing the provision of older-adult care products such as older adult-oriented equipment or accessories, and delivering quality older-adult care services are crucial to promoting the development of older-adult care industry.

Keywords in Cluster 2 include “smart older-adult care,” “intelligence,” “big data,” “informatization,” “Internet,” etc. Hence, Cluster 2 is labeled as “technological support,” which is one of the policy tools. It indicates that policy texts in Cluster 2 focus on the application of information technology and intelligent software products such as artificial intelligence, big data, cloud computing, and blockchain in the field of older-adult care industry. This indicates that the new older-adult care mode driven by technology has become an essential means of solving China's aging problem.

Based on the keywords in Cluster 3, which include “finance,” “fund,” “tax revenue,” “discount,” “capital,” etc., “fiscal and tax support” is an appropriate label for this cluster. Through issuing the incentive policies on tax and finance for the older-adult care enterprises, governments can facilitate the development of older-adult care related enterprises and provide them with financial and tax support.

Cluster 4 includes the keywords like “medical care,” “cure,” “nursing,” “integrated,” “recovery,” etc., so the corresponding policy tool is summarized as “combination of medical care and nursing.” Since the mode of separation of medical care and nursing can no longer satisfy the older adult's' needs, the policy tool “combination of medical care and nursing” is one of the fundamentals of older-adult care industry and makes a contribution to the exploration of the rational allocation of medical care and nursing resources.

Cluster 5 contains keywords such as “professional,” “talents,” “skills,” “training,” and so forth, indicating that the policy texts in this cluster emphasize the professional training. Therefore, the policy tool for this category is named as “professional training.” The aim of the policy tool is to facilitate high-quality development of the older-adult care industry by cultivating excellent professionals with great older-adult care skills.

Cluster 6's keywords include “policy,” “regulation,” “detailed rules,” “environment,” “supporting,” etc. Hence, Cluster 6 can be summarized as “the supporting policy environment.” Sound and thoroughly supportive policies are necessary for the older-adult care industry's development. A sound supporting policy environment includes all relevant policies, regulations, and systems that support the older-adult care industry's development, such as regulations for financial management, measures for the training of older-adult care industry employees, access regulations for older-adult care industry projects, and so forth.

Cluster 7 contains keywords like “demonstration,” “leading enterprises,” “guide,” “brand,” etc., so it is designated as the “pilot projects” policy tool. Promoting the pilot or demonstration projects in the older-adult care industry, and leveraging the leading role of key older-adult care projects will play an essential role in facilitating the development of older-adult care industry.

From the keywords in Cluster 8, which include “insurance,” “finance,” “subsidy,” “stimulate,” “consumption,” etc., it can be inferred that the corresponding policy tool aims to enhance the older adult's payment ability by developing older-adult care insurance and older-adult care inclusive financing, so as to actively stimulate the consumer market of older adults and promote the development of the older-adult care industry. Therefore, this category can be named “the consumer market.”

Cluster 9's keywords include “land use,” “land supply,” “guarantee,” “land price,” etc., suggesting that this category's policy tool pays attention to the older-adult care industry's land demand and aims to provide a land guarantee for the development of older-adult care industry. Based on this, Cluster 9 is designated as “land demand.”

Policy tools are specific measures that governments will use to promote the development of older-adult care industry. Through applying the text mining technology to the older-adult care policy texts, the current research extracts nine policy tools. QCA is then employed to explore the combined effect of nine policy tools, which are older-adult care products and services, technological support, fiscal and tax support, combination of medical care and nursing, professional training, the supporting policy environment, pilot projects, the consumer market, and land demand.

### 4.2 Result variable

The older-adult care industry is a comprehensive industry that comprises primary, secondary, and tertiary industries. In the existing research, there is no unified standard to measure the development of older-adult care industry. As previously mentioned, the term “older-adult care industry” refers to all commodity producers and service providers that meet the older adult's physical and spiritual needs. Based on this definition, the current research takes the number of older-adult care related enterprises across 31 regions in China as an indicator to measure the development of older-adult care industry. Simply put, in the current paper that investigates policy tool mix, the number of older-adult care related enterprises in each region serves as the result variable. Given that the policy effect has a time lag of 2 years and the collected older-adult care policies are issued in 2020 or earlier, the number of older-adult care related enterprises in 2022 is taken as the result variable. Enterprise check software (qcc.com) is used to obtain the number of older-adult care related enterprises across 31 regions. Data of result variable are given in Table 2.

**Table 2:** Number of older-adult care related enterprises in 31 regions.

**Number**	**Region**	**Number of older-adult care-related enterprises in 2022**
1	Jiangsu	28,472
2	Henan	14,171
3	Guangdong	23,929
4	Shandong	23,876
5	Zhejiang	11,594
6	Anhui	13,245
7	Guizhou	9,673
8	Hebei	10,416
9	Sichuan	14,159
10	Jilin	6,808
⋮	⋮	⋮
30	Hunan	10,783
31	Hubei	7,801

### 4.3 Variable assignment

It is necessary to assign values to conditional variables and the result variable after extracting the policy tools through text mining. Since the current research adopts csQCA to explore the combined effects of various policy tools, each variable is assigned a value of either zero or one to facilitate csQCA. Variable assignment is shown in Table 3.

**Table 3:** Variable assignment.

**Variables**	**Description for judgement**	**Value assignment**
Older-adult care products and services (P1)	Older-adult care policy mentions the supply of older-adult care products and services. For example, the policy texts mentioned that to provide multi-level older-adult care services, all parties should vigorously develop older-adult care-related accessories and establish older-adult care related institutions	1
	Older-adult care policy does not mention the supply of older-adult care products and services	0
Technological support (P2)	Older-adult care policy mentions the application of information technologies such as artificial intelligence, the Internet of Things, and big data in the form of, for instance, a smart older-adult care	1
	Policy does not mention the application of information technologies to older adult care	0
Fiscal and tax support (P3)	Policy stipulates that qualified older-adult care-related enterprises should receive financial and tax support. For example, qualified older-adult care enterprises shall receive preferential treatment regarding value-added tax and income tax	1
	Policy text does not mention relevant fiscal and tax support	0
Combined medical care and nursing (P4)	Policy mentions combined medical care and nursing. For example, the policy aims to promote the integration of older adult care services and medical care and establish a stable relationship between older-adult care institutions and medical care institutions	1
	Policy does not mention combined medical care and nursing	0
Professional training (P5)	Policy mentions training for older adult care professionals. For example, the policy acknowledges that training enhances professionals' older adult care skills	1
	Policy does not mention training for older adult care professionals	0
Supporting policy environment (P6)	Supporting policies are mentioned, for example, the establishment of statistical systems and tax regulations	1
	Supporting policies are not mentioned	0
Pilot projects/demos (P7)	Policy acknowledges the importance of pilot projects/demos, for example, in content concerning cultivating leading enterprises and building brands	1
	Policy does not mention pilot projects/demos	0
Consumer market (P8)	Policy includes specific measures for enhancing the older adult's payment ability and cultivating an older adult consumer market, for example, through older-adult care insurance, older-adult care inclusive financing, and so forth	1
	Policy does not mention specific measures for cultivating an older adult consumer market	0
Land demand (P9)	Policy content concerns land demand	1
	Policy does not address land demand	0
Number of older-adult care-related enterprises (N)	Median or above	1
	Below median	0

After the variable assignment, each case which is represented as a combination of conditional variables and result variable should be coded. Through judging whether the older-adult care policy texts in each case incorporate certain policy tools and comparing the number of older-adult care enterprises in the case with the average level, coded cases which is represented as binary data is listed in Table 4.

**Table 4:** Coded cases.

**Case**	**P1**	**P2**	**P3**	**P4**	**P5**	**P6**	**P7**	**P8**	**P9**	**Result variable**
Jiangsu	1	1	0	1	1	1	0	1	0	1
Hunan	1	0	1	1	0	1	0	0	1	0
Shandong	1	1	0	1	0	1	1	0	0	1
…	…	…	…	…	…	…	…	…	…	…

A truth table is then constructed based on Table 4 to clarify the relationship between combinations of conditional variables and result variable. Following Fiss's (52) suggestions, the consistency threshold is set as 0.8, and the case threshold is set as 1. A truth table without contradictory configurations is obtained using the QCA analytics software of fsQCA 3.0.

## 5 Results and discussions

### 5.1 Necessity

**Figure 3:**
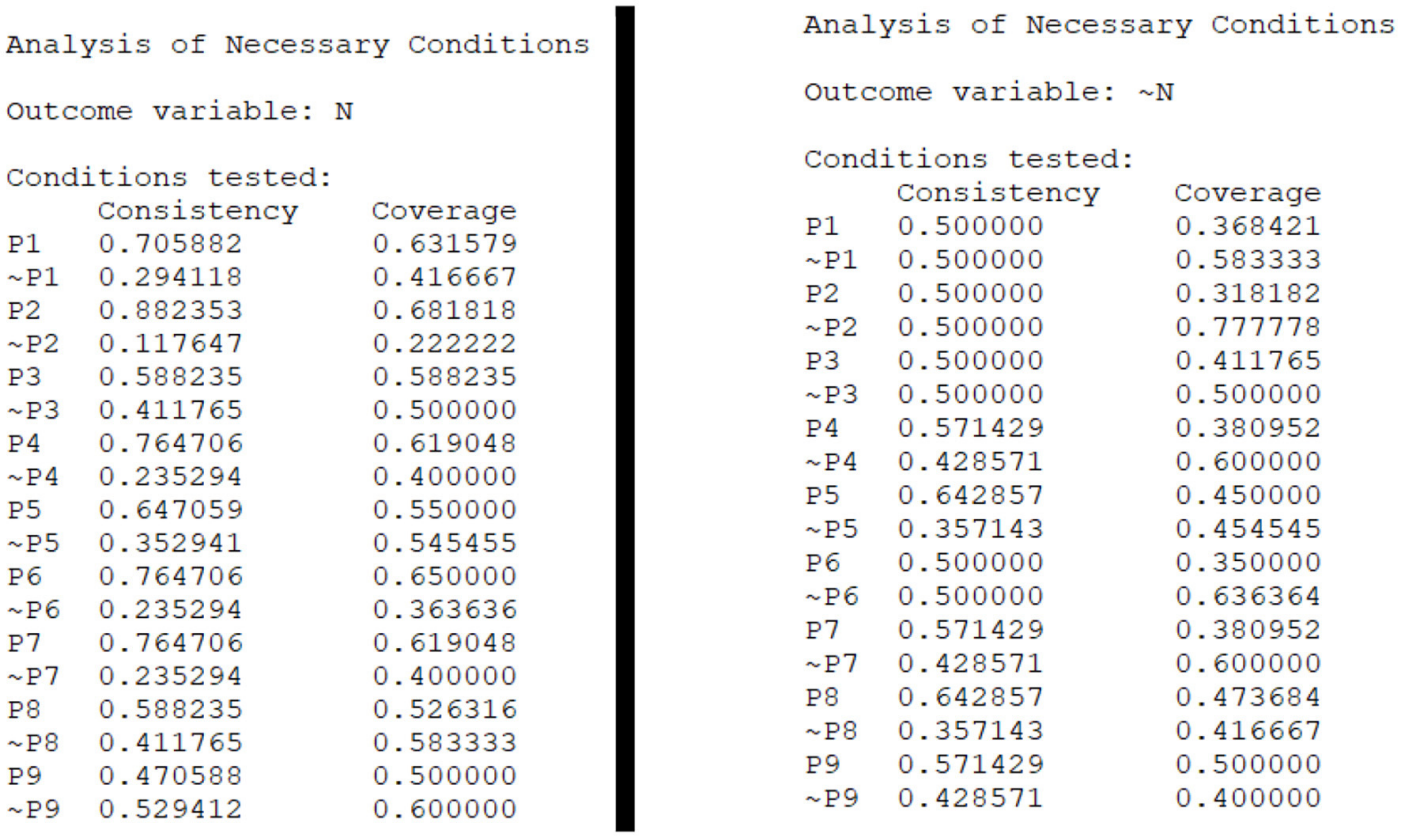
Necessity analysis.

Before analyzing the configurations of nine policy tools, it is important to figure out whether a single policy tool is a necessary condition for improving the development of older-adult care industry, that is, increasing the number of older-adult care related enterprises (53). As long as the consistency level of a condition is >0.9 (49), it can be regarded as a necessary condition to produce the result variable. The number of older-adult care related enterprises (high = N; non-high = ~N) is taken as the result variable, and fsQCA 3.0 software is used to conduct the necessity analysis of the single conditional variable. The results of necessity analysis are displayed in Figure 3, and there is no value reaching the minimum threshold 0.9, which indicates that there is no single policy tool (among P1–9) can be regarded as a necessary condition for the development of older-adult care industry. According to the notion of set theory, it's necessary to further explore the combined effect of nine older-adult care policy tools.

### 5.2 Sufficiency

After performing the necessity analysis, the next step is the configuration anlysis of nine conditional variables. This section is dedicated to investigating the combined effect of nine policy tools on the development of older-adult care industry. High number of older-adult care enterprises is the desired outcome of this research, representing the rapid development of older-adult care industry. Using fsQCA3.0's *standard analysis* function, with the high number of older-adult care related enterprises as the result, three solutions can be obtained based on the truth table, which are complex, parsimonious, and intermediate solutions. The intermediate solution generally lies between the complex and parsimonious solutions. Its conclusion is the most universal and enlightening. Therefore, the intermediate solution is the most representative explanation and is usually considered to be the solution that can best explain the problem (54). Notably, when the conditional variable appears simulataneously in both the intermediate and parsimonious solutoins, it is regarded as the core condition, which means that the variable decisively impacts the result. When the conditional variable only appears in the intermediate solution and not in the parsimonious solution, it is regarded as the edge codition, which plays an auxiliary role.

This study ultimately derives five configurations of older-adult care policy tools to promote the older-adult care industry's development. Policy tool mixes are displayed in Table 5 as R1–5.

**Table 5:** Configurations of policy tools to promote the development of older-adult care industry.

**Conditional variables**	**Configurations of policy tools to promote the development of the older-adult care industry**				
	**R1**	**R2**	**R3**	**R4**	**R5**
P1	⊗	•	⊗	•	•
P2	•	•	•	•	⊗
P3		•	•	⊗	**•**
P4	•	•		•	•
P5	⊗	•	•	•	
P6	•	⊗	•	•	•
P7	⊗	⊗	⊗	⊗	
P8		⊗	•		•
P9	⊗		⊗	⊗	•
Consistency	0.97	0.93	0.90	0.87	0.86
Raw coverage	0.27	0.29	0.15	0.41	0.22
Unique coverage	0.11	0.05	0.16	0.22	0.16
Solution consistency	0.91				
Solution coverage	0.65				

• indicates a core condition, • indicates an edge condition, ⊗ indicates the absence of core conditions, and ⊗ indicates the absence of edge conditions. A blank space indicates that the condition is optional. The same applies to Table 6.

### 5.3 Discussions

Reading Table 5 horizontally, it is evident that among the five configurations, combination of medical care and nursing (P4) is the core condition in four configurations. Therefore, combination of medical care and nursing is an essential policy tool to promote the development of older-adult care industry. Among the older-adult care policy texts collected from 31 regions, all of them involve the requirements and regulations for the promotion of combination of medical care and nursing. By contrast, four configurations in R1–R5 lack the policy tool of pilot projects (P7), indicating that this policy tool does not play a significant role in promoting the development of older-adult care industry.

In a vertical reading of Table 5, the application of the nine policy tools across all configurations (R1–5) differs, but all five configurations promote older-adult care industry. Based on the characteristics of the policy tools utilized in each configuration, we summarize five policy tool mixes:

- Supporting policy-driven mode. In the first policy tool configuration R1, the core conditions are technological support (P2) and combination of medical care and nursing (P4), with the absence of older-adult care products and services (P1), pilot projects (P7), and land demand (P9), while the edge condition is the supporting policy environment (P6), with the absence of professional training (P5). That is, in R1, technological support and combination of medical care and nursing are the core policy tools, while the supporting policy environment is the auxiliary condition and thus an indispensable policy tool for older-adult care industry's development. Therefore, R1 is labeled as “supporting policy-driven mode.” As an important older-adult care policy tool, supporting policy environment plays a crucial role in promoting older-adult care industry development. Specifically, the development of older-adult care industry relies on various supporting policies such as taxation, evaluation, supervision, etc. Only by establishing sound and comprehensive supporting laws and regulations can we facilitate the older-adult care industry.
- Fiscal and tax support mode led by supply-oriented policy tools. In R2, the core conditions are older adult care products and services (P1), technological support (P2), combination of medical care and nursing (P4), and professional training (P5), with the absence of pilot projects (P7) and consumer market (P8), while the edge condition is fiscal and tax support (P3), with the absence of supporting policy environment (P6). This configuration indicates that in regions that lack a supporting policy environment, pilot projects, and a consumer market of older adults, the development of older-adult care industry depends primarily on older adult care products and services, technological support, combination of medical care and nursing, professional training, and fiscal and tax support. Among these, older adult care products and service, technological support, combination of medical care and nursing, and professional training are, as the core policy tools, the driving forces of older-adult care industry. With reference to the research of Zhu et al. (59), they are supply-oriented policy tools. As an auxiliary condition, fiscal and tax support, classified as an environmental policy tool, is necessary to sustain the development of older-adult care industry. Therefore, R2 is labeled “the fiscal and tax support mode led by supply-oriented policy tools.” This configuration shows that enhancing the older-adult care industry requires fiscal and tax support, as well as the supply-oriented policy tools such as older adult care products and services.
- Double-team mode driven by fiscal and tax support and the consumer market. In R3, the core conditions are fiscal and tax support (P3) and the consumer market (P8), with the absence of older adult care products and services (P1) and pilot projects (P7), while the edge conditions are technological support (P2), professional training (P5), and a supporting policy environment (P6), with the absence of land demand (P9). Current research has found that this configuration has obvious double-team driving characteristics, that is, fiscal and tax support and consumer market jointly play a positive role in promoting the older-adult care industry. On the one hand, the development of consumer market can improve the older adult's payment ability through older-adult care insurance, older-adult care financing, and so forth. With reference to the research of Zhu et al. (59), the consumer market is a demand-oriented policy tool that targets at older adults as consumers, while the fiscal/tax support provides essential financial support for older-adult care related enterprises and institutions. Both the older adult consumer market and fiscal and tax support jointly contribute to the development of the older-adult care industry.
- Multi-subject joint force mode driven by older-adult care products and services, technological support, and combined medical care and nursing. In R4, the core conditions are older-adult care products and services (P1), technological support (P2), and combination of medical care and nursing (P4), with the absence of fiscal and tax support (P3), pilot projects (P7), and land demand (P9), while the edge conditions are professional training (P5) and a supporting policy environment (P6). In this configuration, the policy tools of older-adult care products and services, technological support, and combination of medical care and nursing must be adopted together. The reason underlying the integrated application of these three policy tools is that as a new older-adult care mode, combination of medical care and nursing involves in a variety of smart medical equipment, that is, technological support to provide the older adults with advanced older-adult care products and services such as daily care, medicine rehabilitation, etc. The three policy tools complement each other to jointly promote older-adult care industry. In this path, to promote the development of the older-adult care industry, attention should be paid to the deep application of information technology in the older-adult care field while vigorously developing the combination of medical care and nursing and enriching the supply of older-adult care products and services (56).
- Technology compensation mode. In R5, the core condition is combination of medical care and nursing (P4), with the absence of technological support (P2), while the edge conditions are older-adult care products and services (P1), fiscal and tax support (P3), supporting policy environment (P6), consumer market (P8), and land demand (P9). In this configuration, despite the lack of technological support, the other policy tools adequately promote the development of older-adult care industry. This means that the core condition of combination of medical care and nursing and the five edge conditions, which are older adult care products and services supply, fiscal and tax support, supporting policy environment, consumer market, and land demand, can compensate for the lack of technological support, to a certain extent.

### 5.4 Robustness test

With reference to the research of Du and Jia (49), the current research adopts robustness test. The low number of older-adult care-related enterprises (~N) is taken as the result variable, and six policy tool configurations that impede the development of older-adult care industry are generated, as shown in Table 6.

**Table 6:** Configurations of policy tools to impede the development of older-adult care industry.

**Conditional variables**	**Policy tool configurations that would impede older-adult care industry development**				
	**R1**	**R2**	**R3**	**R4**	**R5**	**R6**
P1	⊗	•			⊗	
P2	⊗	•		•	⊗	
P3	⊗	⊗	•	•	⊗	
P4	⊗	⊗	•			⊗
P5	•	⊗	•	⊗		⊗
P6	•		⊗	⊗	•	⊗
P7		⊗	⊗	•		•
P8		⊗	⊗	⊗	•	•
P9	•			⊗	•	•
Consistency	0.91	0.91	0.92	0.88	0.90	0.89
Raw coverage	0.33	0.31	0.25	0.40	0.22	0.05
Unique coverage	0.33	0.11	0.25	0.15	0.22	0.05
Solution consistency	0.93					
Solution coverage	0.86					

Through comparing the configurations of policy tools in Tables 5, 6, it can be learned that there are obvious differences between configurations promoting older-adult care industry and configurations impeding it. Notably, however, the configurations of policy tools that promote the development of older-adult care industry are not simply the opposites of configurations that would impede it, indicating that asymmetry in the result variables' causal factors. Therefore, the research results are relatively stable.

## 6 Conclusions and implications

### 6.1 Conclusions

A scientific policy tool mix is of vital significance to the development of older-adult care industry. To facilitate the older-adult care industry in China, the current research employes the combination of text mining technology and QCA method to address the question of “How do policies promote the sustainable development of older-adult care industry?” Specifically, we extract the policy tools through text mining the older-adult care policies of 31 provinces, autonomous regions, and municipalities in China. Then with the goal of promoting the development of older-adult care industry, we explore the combined effect of various older-adult care policy tools from the configuration perspective, and derive five older-adult care policy tool mixes through QCA method. The main findings and conclusions which address the corresponding research questions proposed in Introduction section are shown below:

- Through applying the K-means clustering and the TF-IDF algorithm, nine older-adult care policy tools are extracted from the policy texts, namely older-adult care products and services, technological support, fiscal and tax support, combination of medical care and nursing, professional training, the supporting policy environment, pilot projects, the consumer market, and land demand.
- By conducting the necessity analysis and sufficiency analysis, it can be concluded that the development of older-adult care industry is driven by the combined effect of multiple policy tools, not a single policy tool. Among the policy tools of older-adult care products and services, technological support, fiscal and tax support, combination of medical care and nursing, professional training, supporting policy environment, pilot projects, consumer market, and land demand, none of them can constitute the necessary condition for promoting older-adult care industry separately.
- Five policy tool mixes that promote the development of older-adult care industry are identified, which are supporting policy-driven mode, fiscal and tax support mode led by supply-oriented policy tools, double-team mode driven by fiscal and tax support and the older adult consumer market, multi-subject joint force mode, and technology compensation mode. Although each policy tool mix differs in terms of the policy tools utilized and their configuration, all five mixes promote the older-adult care industry, indicating the equivalency among the policy tool mixes. Furthermore, the results of robustness test show that the policy tool mixes that promote the development of older-adult care industry are not merely the opposite of those configurations that would impede older-adult care industry, indicating the asymmetry among the policy tool mixes.

## 7 Implications

With the application of text mining and QCA method, the configurations and combined effect of various policy tools are derived, and the following implications for theory, policy and practices are derived:

- In terms of the implications for theory, the application of text mining technology and QCA method to explore the policy tool mix contributes to the development of contemporary policy sciences. The “tools approach” to policy studies in which the outputs of the policy process are conceptualized as comprised of specific policy instruments or policy tools deployed in the effort to achieve specific policy objectives (55). Understanding the uses of these “portfolios” or “bundles” of instruments is a focus of mech current interest in tool studies which contributes to help design, promote and implement more effective governance strategies. To our best knowledge, this paper is the first attempt to combine text mining and QCA method to investigate the “portfolios” or “bundles” of policy tools in the field of older-adult care, leading to the progress of the contemporary policy sciences.
- In terms of the implications for older-adult care policy and practices, the policy practitioners should pay attention to the combined application of policy tools. Based on the policy tool mixes identified in this research, the integration of different policy tools should be emphasized. To improve the older-adult care industry, local governments should take into account their actual situation and existing resources, and select the most suitable configurations of policy tools for their unique developmental realities.

In practice, the application of combination of medical care and nursing should be strengthened, given that it's a key policy tool to promote the development of older-adult care industry. It is an innovative older-adult care mode that integrates the resources of medical care and nursing to provide the older adults with continuous and targeted medical and nursing services. Since the traditional older-adult care mode supported by family is weakening, and the “bed pressure” in hospitals for the older adults is prominent, the combination of medical care and nursing is an inevitable trend in China (57, 58). Different from the traditional home-based care or community-based care, the combination of medical care and nursing can introduce the medical resources into nursing care services and provide disease prevention, rehabilitation care, and life care for older adults, realizing the system integration and thus promoting the older-adult care industry.

Additionally, the application of pilot projects should be decreased in the practice of older-adult care policy implementation. Configuration analysis of older-adult care policy tools reveal that pilot projects is an auxiliary condition in the policy tool mixes. Hence, the policy tool of pilot projects has little effect on the development of older-adult care industry. Considering the limited resources, governments cannot take into account all policy tools simultaneously, and they need to focus on the core policy tool and take advantage of the limited resources. Hence, to facilitate the development of older-adult care industry, governments should reduce the application of pilot projects to save costs and make the best of limited resources.

Owing to it is the first attempt to study the configurations and combined effect of older-adult care policy tools with the application of text mining and QCA method, there is a limitation in the current research, which is shown below. Since there is no unified standard to measure the development of older-adult care industry, the current research takes the number of older-adult care related enterprises across 31 regions in China as an indicator to measure the development of older-adult care industry, which may not represent the development of older-adult care industry scientifically. In the future, we should explore a more reasonable and effective indicator that can reflect the development of older-adult care industry.

## Data Availability

The raw data supporting the conclusions of this article will be made available by the authors, without undue reservation.
